# Paralympics - Addendum to the Update on the Guidelines for Sport and Exercise Cardiology of the Brazilian Society of Cardiology and the Brazilian Society of Exercise and Sports Medicine

**DOI:** 10.5935/abc.20190194

**Published:** 2019-09

**Authors:** Japy Angelini Oliveira Filho, Antônio Claudio Lucas da Nóbrega, Luiz Gustavo Marin Emed, Marcelo Bichels Leitão, Roberto Vital

**Affiliations:** 1 Universidade Federal de São Paulo - Escola Paulista de Medicina, São Paulo, SP - Brazil; 2 Universidade Federal Fluminense, Niterói, RJ - Brazil; 3 Hospital Cardiológico Costantini, Curitiba, PR - Brazil; 4 Sociedade Brasileira de Medicina do Exercício e do Esporte, São Paulo, SP - Brazil; 5 Universidade Federal do Paraná, Curitiba, PR - Brazil; 6 Comitê Paralímpico Brasileiro, São Paulo, SP - Brazil; 7 Universidade Federal do Rio Grande do Norte, Natal, RN - Brazil

**Keywords:** Sports, Athletes/history, Athletes/legislation &jurisprudence, Disabled Persons, Physical and Rehabilitation Medicine, Hearing Loss, Vision Disorders

## Para-athletes or athletes with disabilities

Paralympic sports include a wide group of athletic activities for individuals who have disabilities and who participate in diverse levels of competition.

The Paralympic Movement began in 1888 in Berlin with the founding of the first sports clubs for people with hearing impairments.

In 1922, the International Committee of Sports for the Deaf (CISS) was founded and the First International Silent Games were organized.

In 1989, the International Paralympic Committee was founded in Dusseldorf (http://www.paralympic.org), related to diverse federations connected to athletes with disabilities.

In 1945, Ludwig Guttmann, a doctor specializing in neurological surgery, initiated rehabilitation programs for World War II veterans with special needs at the National Spinal Injuries Centre of the Stoke Mandeville Hospital, in England. The first competition took place with 16 war veterans, on July 29, 1948, during the Opening Ceremony of the 1948 Summer Olympics in London. In 1960, the first Paralympic Games took place in Rome, with 400 athletes from 23 countries; on that occasion, Pope John XXIII referred to Guttmann as “the de Coubertin of the paralyzed.” Since the Summer Games of 1988 and the Winter Games of 1992, the Paralympics have been held in the same city as the Olympics.

In Brazil, Paralympic sports began in 1958, when the wheelchair user Robson Sampaio de Almeida and the physical trainer Aldo Miccolis founded the Clube do Otimismo. Shortly thereafter, Sérgio Seraphin Del Grande, an athlete with a disability, founded the Clube dos Paraplégicos de São Paulo. The National Association for Parasports (Associação Nacional de Desporto de Deficientes [ANDE]) was founded in 1975.

In 1995, the Brazilian Paralympic Committee (http://www.cpb.org.br) was founded. Its headquarters, initially located in the city of Niterói, Rio de Janeiro State, moved to Brasília in 2002. The Brazilian Paralympic Committee has the following vision, mission, and principles:

“1 - **Vision:** to represent and lead the Brazilian Paralympic Movement, seeking to promote and develop high-performance sports for people with disabilities;
2 - **Mission:** To exercise the legitimate representation of Brazilian Paralympic sports; To organize Brazil’s participation in continental and worldwide competitions and in the Paralympic Games; To promote the development of diverse Paralympic sports in Brazil, in conjunction with the respective national organizations; To promote universal access of people with disabilities to athletic practice on their diverse levels;
3 - **Principles:** To work in full partnership with technical areas of national associations and confederations affiliated and connected to the Brazilian Paralympic Committee, valorizing the convergence of objectives in favor of the development of every segment of Brazilian Paralympic sports.”

Paralympic Medicine deals with healthcare related to athletes with disabilities.^1^ As of 1994, the International Paralympic Committee, aims to bring scientific support to the Paralympics, without interfering with athletes, training, and organization of the games.^2^ There are 4 core Paralympic values established by the International Paralympic Committee. They are courage, determination, inspiration, and equality.^3^

The development of specialized prostheses and equipment, such as specific wheelchairs is essential for the optimized use of parathletes’ residual mechanical function.^4,5^ Two-dimensional kinematic analysis of athletic gestures may be useful, given the broad diversity of parathletes’ residual functional capabilities.^6^ Furthermore, sports psychologists should assist parathletes in developing mental skills for stress management and consequent improvements in athletic performance.^7^

### Cardiological evaluation: pre-participation and re-evaluation

All Paralympic athletes should undergo evaluation, regardless of age, sex, and associated disability; pre-participation evaluation should include male and female children, adolescents, adults, and masters/elderly athletes, and it should be the sole responsibility of the attending physician (class of recommendation: I, level of evidence: C).

Evaluation should be comprehensive, considering the organism as a whole, with emphasis on physical and somatic aspects; it is necessary to bear in mind that, in physical training and athletic performance, there are interactions between physical disabilities, comorbities, and their respective sequelae (class of recommendation: I, level of evidence: C).

Frequency of re-evaluation should be at the discretion of the attending physician, in accordance with each case’s characteristics; the primary aim of re-evaluation frequency should be safe athletic practice (class of recommendation: I, level of evidence: C).

Evaluation of Paralympic athletes must follow the established protocol, which is summarized in Table 1.

**Table 1:** Protocol for evaluating Paralympic athletes, according to the Medical Department of the Brazilian Paralympics Committee (http://www.cpb.org.br)

1. Application of a standardized medical questionnaire, involving identification, personal and family history, sports history, and dietary and daily living habits;
2. Physical examination, with standardized medical form;
3. Laboratory exams: complete blood count, iron, ferritin, folic acid, vitamin B12, blood type, total lipids, cholesterol and fractions, triglycerides, uric acid, blood glucose, type I urine, creatinine, urea, sodium, potassium, testosterone, free testosterone, insulin, cortisol, free T4, free T3, T3, T4, TSH, serology for Chagas, herpes, HIV and HCV, total proteins, AST, ALT, GGT, alkaline phosphatase, calcium, and homocysteine;
4. Chest radiograph;
5. Resting ECG and ergometric test.

Following initial evaluation, based on the findings, specialized exams will be indicated at the attending physician’s discretion. Examples include cardiopulmonary exercise test (CPT), echocardiogram (ECHO), vectorcardiogram (VCG), computerized tomography, magnetic resonance, ultrasound, hemoglobin electrophoresis (to investigate sickle cell anemia), cardiological evaluation, ophthalmic evaluation, (to investigate Marfan syndrome, glaucoma, and retinal detachment), and orthopedic evaluation^8,9^ (class of recommendation: I, level of evidence: C).

For athletes with cerebral palsy, the spasticity assessment score (quantitative sports and functional classification [QSFC]) may be used, based on muscular conditions in the upper and lower limbs and the torso. The score may be used for clinical investigation, clinical treatment, and physical training.^10^

Athletes who use wheelchairs or prostheses should be closely and thoroughly examined for decubitus sores or sores in the region of the prosthetic implant. The presence of ulcers in these areas will make the athletes temporarily ineligible, until the local conditions of the tegument have been restored (class of recommendation: I, level of evidence: C). The practice of urinary retention should be prohibited in athletes who use wheelchairs, owing to the risk of high elevations in blood pressure and stroke. In cases of neurogenic bladder, it is necessary to pay attention to the presence of subclinical urinary infection.

The occurrence of athletic heart syndrome, considering the presence of 2 or more signs, affected 46% of Paralympic athletes. Signs of athletic heart syndrome occurred in 33% of clinical exams (murmurs and clicks), in 55% of electrocardiograms (bradycardia, incomplete right bundle branch block, overloads, T-wave alterations), in 15% dos vectorcardiogram (overloads), and in 5% of echocardiograms (cavity dimensions higher than normal). Signs occurred in 51% of athletes, with 46% of cases having 2 or more signs and 12% having 4 or more signs. ET was normal in 77% of athletes; ischemic ST segments were not found. Right bundle branch block was present in 23% of cases.^11^

The following ECG alterations, classified as athletic ECG, were found in Paralympic athletes: primary alterations in ventricular repolarization, 6%; first-degree atrioventricular block, 2%; sinus bradycardia, 6%; block of the anterosuperior division of the left His bundle branch, 2%; right His bundle branch conduction disorder, 14%; early ventricular repolarization, 29%; left atrial overload, 2%; left ventricular overload, 39%.^12^

Eight cases of late potentials were described on high-resolution electrocardiogram in 11% of athletes, and there was no evidence of heart disease in a consecutive series of 79 top athletes with disabilities.^13^

In subgroups of Paralympic athletes, significant correlations have been described involving variables related to aerobic potential, anaerobic threshold, and morphological variations evaluated by echocardiogram, proving that athletic heart syndrome may occur in Paralympic athletes.^14^

In Paralympic Judo athletes, the presence of athletic heart syndrome has been found in 64% of cases evaluated.^15^

In subjective evaluations of young Paralympic athletes, it was found that parents might report lower perceived quality of life than their children.^16^

### Cardiopulmonary exercise test

The bases of cardiopulmonary exercise testing protocols include: 1) reproducibility of athletic actions, according to the principle of specificity; 2) adequacy for the athletic modality and the athlete’s means of locomotion; 3) performance of tests with stability and safety, guaranteeing accuracy and reproducibility of measurements^11^ (class of recommendation: I, level of evidence: B).

Special care should be taken in relation to type and degree of disability, the athlete’s posture, room temperature, prior emptying of bladder, prevention of hypertension, the risk of seizures and accidents, blood pressure measurements, and adequate mask sealing. Numerous factors may limit evaluation performance, including: 1) clinical factors: mental and sensorial disabilities (visual, tactile, or hearing impairments, epilepsy, autonomic dysreflexia, neurogenic bladder, sympathetic deprivation, post-polio syndrome, stress-induced tachypnea, malnutrition); 2) locomotive factors: reduced body mass, reduced muscle strength and flexibility, increased muscle tone, reduced joint mobility, reduced motor coordination, osteoarticular injuries secondary to athletic practice, amputation stump injuries; 3) cardiovascular factors: eventual associated infections; 4) physiological factors: reduced peak VO_2_, anaerobic threshold, respiratory compensation point, early fatigue, physical inactivity; 4) socioeconomic and cultural factors: social exclusion, lack of funds.^8^

According to the principle of specificity, arm ergometers have been used for tennis players, throwers, weightlifters, fencers, and swimmers; bicycles for cyclists; and treadmills for all other modalities;^8^ cyclists may use their own equipment, attached to a Mag 850 Minoura 180 system (class of recommendation: I, level of evidence: C).

Athletes should be encouraged to reach their “real” maximum effort. Athletes with cerebral palsy or mental disabilities require prior, detailed explanation regarding the tests, owing to inherent challenges in comprehension. It is possible to carry out the first exams of the day with athletes who have been previously evaluated on other occasions, allowing those who will undergo the test for the first time to watch and understand the execution and objectives of the test.^17^

Some precautions should be followed. In athletes with cerebral palsy, there exists a predisposition to accidents (falling), in treadmill tests, due to lack of neuromuscular coordination, especially at higher velocities. In some cases, it is necessary to increment workload using inclination rather than velocity. Due to involuntary facial movements or very acute mandibular angle, the mask or mouthpiece may not seal adequately, and escape may occur in uptake of gases. Athletes with visual impairments, in most cases, will need to maintain contact with their hands on the rail of the treadmill (without leaning on it), and they will need to receive verbal orientation on their biomechanics and spatial situation. Safety belts, which are tied to the athlete’s waist and to the front rail of the treadmill, may be used in some cases.^17^

Cardiopulmonary exercise tests may be carried out following numerous specific protocols, examples of which are summarized in Table 2.

**Table 2:** Protocols for cardiopulmonary exercise tests for Paralympic athletes (Centro de Estudos em Fisiologia do Exercício - Unifesp/Escola Paulista de Medicina)^8^

Wheelchair treadmill CET	Initial velocity of 3 to 13 km/h and initial inclination of 0 to 2%, with increments of 0.5 to 1.0 km/h and 0.5 to 1.0% every 3 minutes
Treadmill CET	Initial velocity of 3 to 8 km/h and initial inclination of 0%, with increments of 0.5 to 1.0 km/h and 0.5 to 5.0% every 3 minutes
Exercise bicycle CET	Initial load of 25 to 50 watts, with increments of 25 watts every 3 minutes
Roller bicycle CET	Initial velocity of 30 to 33 km/h, with increments of 3 km/h every 3 minutes
Arm ergometer CET	Initial load of 25 a 37.5 watts, with increments of 5 to 25 watts every 3 minutes

CET: cardiopulmonary exercise test

Knechtle and Köpfli’s^18,19^ Protocol (Institute of Sports Medicine, Swiss Paraplegic Centre) for wheelchair treadmill testing begins at a velocity of 8 km/h and an inclination of 1%, with 0.5% increments every 2 minutes and constant velocity, until exhaustion.^18,19^

There are also advantages to carrying out field tests.^20^ Variations between 48% and 80% have been described in regression equations for determining physical capacity in people with paraplegia and quadriplegia. These variations may be explained by the level and degree of spinal cord injury, age, gender, physical activity, and body weight. In Brazil, values referring to aerobic potential of Paralympic athletes have been similar (Table 3).^21^

**Table 3:** Aerobic potential of Brazilian Paralympic athletes participating in the Atlanta Games. Silva AC, Torres FC, Oliveira Filho JA. Avaliação dos atletas paraolímpicos de Atlanta. Unpublished data. Unifesp-EPM, São Paulo, 2006^22^

Modality/disability	n	VO_2 max_ ml.kg^-1^.min^-1^	Variation ml.kg^-1^.min^-1^	LA %
Football ♂ CP	18	50.6 ± 6.70	36.5 - 62.8	70 ± 9
Swimming ♂ tetra, PM, SCI	7	36.8 ± 17.7	19.8 - 59.0	64 ± 5
Swimming ♀ para, PM, SCI	4	48.9 ± 9.90	35.3 - 61.4	56 ± 9
Basketball ♀ PM, SCI, amp	14	30.0 ± 6.00	20.0 - 40.0	61 ± 8
Tennis ♂ SCI	2		29.7 - 33.3	60
Table tennis ♂ SCI, PM	2		31.0 - 34.5	64.67
Judo ♂ VD	4	45.5 ± 12.0	36.0 - 62.0	59 ± 11
Field/wheel ♂ tetra, PM, CP	3	32.8 ± 10.0	25.0 - 44.0	60 ± 2.9
Field/wheel ♂ para, amp	2	39.0 - 42.0		47.62
Track ♂ VD	3	57.0 ± 7.0	50.0 - 65.0	80 ± 5
Track ♀ VD	2		51.0 - 59.0	46.72
Pentathlon/wheel ♂ para, PM, amp	2		44.0 - 51.0	64.81

amp: amputation; AT: anaerobic threshold; CP: cerebral palsy; para: paraplegia; PM: poliomyelitis; quad: quadriplegia; SCI: spinal cord injury; wheel: wheelchar; VD: visual disability

Pre-participation evaluation for leisure activities is similar, depending on physical and mental stress. In many situations, given the emotional burden involved and the lower level of training, physical and psychological stress may be highly intense, to a degree similar to that of competitions.

### Preventing injuries/sudden death in sports

Injury prevention should include prevention of accidents, aggravation of pre-existing injuries and comorbidities, and sudden death.

The objectives of an athletic injury prevention protocol are based on pre-participation screening:

1. Identification of predisposing conditions, or be it, cardiovascular diseases that may potentially cause sudden death;
2. Definition of measures that may be taken to reduce risk of sudden death: “What are they?” “How should they be developed?”
3. Standardization approach to be adopted for each heart disease and discussion of the eventual disqualification of an athlete to exercise his or her profession.

Prevention of injuries and sudden deaths in sports and leisure activities is carried out considering early diagnosis and treatment of cardiovascular disorders, as well as the application of up-to-date ineligibility criteria, which are duly applied to Paralympic athletes.^23^ It is imperative that competition venues have medical and paramedical resources that are properly equipped for emergency response.

In various institutions, the clinical director and/or the attending physician responsible are accountable to the respective Regional Council of Medicine regarding compliance with these norms.

Individuals who have been recently hospitalized or who have been sedentary for a long time will require progressive training with gradual increments in exercise intensity and session frequency and duration; in addition to the risks which physical injuries and sequelae present, their appearance may be a factor that discourages training, negatively impacting self-image and predisposing athletes to abandon the program (class of recommendation I, level of evidence C).

### Ethical Aspects

Medical evaluation should include specialists from diverse areas, with exercise and sports medicine, cardiology, orthopedics, and physiatry standing out.

When treating athletes with disabilities, it is important to emphasize that it is the physician’s exclusive competence to direct training, diagnose any eventual pathologies and sequelae, request exams, prescribe therapy, and remove athletes from athletic activities; the doctor is not allowed to delegate functions within his or her exclusive competence to individuals who are not qualified to practice medicine (Brazilian Federal Council of Medicine, Resolution nº 1236/87). On the other hand, training should be conducted by physical education instructors and physical therapists. The interaction between doctors, physical education instructors, physical therapists, physiologists, nutritionists, and psychologists is fundamental to a program’s success. Training should be prescribed by means of a medical prescription that states the modality, frequency, and duration of sessions, as well as training intensity and other observations at the attending physician’s discretion. This conduct has been ratified by the Brazilian Federal Council of Medicine Position 4141/2003: “In all of the above, it is the physician’s exclusive competence, following diagnosis of a disease, to prescribe adequate therapy for a patient, including the prescription of physical activity in view of the disease diagnosed or to prevent diverse diseases.”

In various institutions, the clinical director and/or responsible attending physician are accountable regarding compliance with these norms before their respective Regional Council of Medicine. Physicians’ relationships with other professionals in the area of healthcare should be based on mutual respect, freedom, and professional independence for all involved, always seeking the interest and well-being of their patients (Brazilian Code of Medical Ethics, January 1, 1988, Article 18). Interaction between physicians, physical education instructors, physical therapists, nutritionists, psychologists, and trainers is fundamental to a training program’s success, and it should be encouraged at all times.

### Recommendations

Currently, given the scarcity of reports in specialized literature, criteria for attending Paralympic athletes are generally based on specialist consensus (level of evidence: C).

Determination of athletic eligibility should follow the protocol of the International Paralympic Committee Classification Code and International Standards^24^ and the Brazilian Olympic Committee. In this manner, Paralympic athletes may be eligible for one activity and ineligible for another. Athletic eligibility criteria for all Paralympic activities are defined by the respective international federation. Physicians, trainers, and Paralympic athletes should be aware of the risks of eventual and involuntary doping.^25^ Since 2000, an overall incidence of < 1% of violations related to doping has been reported in Paralympic competitions. These are generally detected by urine tests during competition periods, comprising a total of 60 violations, 37 of which were in weightlifters.^26^

Recommendations for attending Paralympic athletes are listed in Table 4. The practices of doping and boosting are to be severely prohibited. Spinal cord injuries lead to changes in autonomic and cardiovascular function, thus interfering with athletic performance. In these cases of spinal cord injuries at or above level T6,^27^ boosting may occur. Boosting intentionally induces autonomic dysreflexia during competition.^28^ For the purpose of obtaining rapid elevations in blood pressure, some athletes who use wheelchairs might induce a state of autonomic dysreflexia, a reflex which occurs in the lower part of the body.^29^ Boosting leads to an increase in circulating catecholamines, blood pressure, and heart rate, and it leads to a 9.7% improvement in racing time for 7% to 10% of athletes.^30^ The practice of boosting, however, exposes athletes to serious risks during competitions, and it is officially banned by the International Paralympic Committee.^30^ In order to obtain a rapid increase in blood pressure athletes who use wheelchairs have been reported to provoke a state of autonomic dysreflexia by exposing the lower part of the body to painful stimuli, which include keeping one’s bladder full, tying belts or straps around one’s legs, sitting on pointed objects, sitting on one’s own scrotum, closing one’s catheter tube in order to fill the bladder, bending one’s feet in the wheelchair, and provoking the fracture of toes.^29^ Only Paralympic athletes with high-level spinal cord injuries may experience episodes of autonomic dysreflexia.^31^

**Table 4:** Recommendations for attending Paralympic athletes (class of recommendation: I, level of evidence: C)

1. All Paralympic athletes should undergo evaluation, regardless of age, sex, and associated disability.
2. Pre-participation evaluation should include male and female children, adolescents, adults, and elderly athletes, and it should be the sole responsibility of the attending physician.
3. Re-evaluation frequency should be at the discretion of the attending physician, in accordance with each case's characteristics; the primary aim of re-evaluation frequency should be safe athletic practice.
4. Evaluations should follow the protocol of the International Paralympic Committee, and they should by specific for each athletic activity and individualized for each athlete.
5. Clinical and cardiological evaluations should be coordinated and carried out by physicians; physical education instructors, physical therapists, physiologists, nutritionists, and psychologists should participate in evaluation, and the integration of physicians and other healthcare professionals is of great value.
6. Clinical evaluation should include all parts and systems of the organism, and it should be performed by a multi-professional team involving diverse medical specialties.
7. Cardiovascular evaluation follows the same general eligibility criteria for athletes without disabilities.
8. Pharmacological prescriptions should always be guided by the WADA's latest policies, which are periodically updated.

WADA: World Anti-Doping Agency

During medical care, special attention should be paid to musculoskeletal injuries, which accounted for 44.6% of 2,590 accredited medical encounters.^32^ Considering any musculoskeletal complaint which led athletes to seek medical attention, the occurrence of sports injuries during the Winter Paralympic Games was 9.4% in 2002, 8.4% in 2006, and 24% in 2010. This proportion was similar in men (22.8%) and women (26.6%).^33^
